# 

**Published:** 1906-03-15

**Authors:** 


### ANNOUNCEMENTS.

DENTAL HINTS DISCONTINUES PUBLICATION.—The January issue of the *American Journal of Dental Science*, published at Madison, Wisconsin, announces that it has taken over the editor of *Dental Hints* and absorbed that journal. As a clearing house for disposing of superfluous dental journals Brother Beecroft has the record, he shouldn't let the *International* with its splendid record, lapse into oblivion without some formal ceremony.

THE WESTERN DENTAL JOURNAL.—The January number of this popular dental journal comes with a new and handsome cover and three new editors to assist Dr. Patterson in supplying the copy for its seventy-six pages of reading matter. It is printed on good paper and the press work is a decided improvement on the old journal. Dr. W. J. Brady will have charge of a department of Orthodontia, Dr. F. O. Hetrick will conduct a miscellaneous department and Dr. F. G. Worthley will supply Laboratory Hints. A formal editorial department is also new and good. We trust it may avoid the "ponderous" in its treatment of editorial matter.

DEATH OF PRESIDENT LEWIS.—Mr. H. M. Lewis, the President of the S. S. White Dental Manufacturing Company, for many years, died January 26, 1906. Any one who knew Mr. Lewis will be very sorry to learn of his death. He was the great mind of that corporation, and one of the most capable business men in the dental supply business. He was universally honored and respected by all the dental trade and many of the profession. He was one of the most kindly and corteous gentleman we ever had the pleasure of meeting, and we cordially sympathize with his family and friends in their great loss.

---

Dental Supply House Fire.—On January 5th, the Hettinger Bros. Dental Depot of Kansas City, was completely destroyed by fire. With characteristic Western energy another dental supply house was bought out and the firm did'nt lose much time in getting started. The mailing list of the *Western Dental Journal* was also destroyed.

---

Recent Patents.—Dr. J. W. Dennis, of Cincinnati, has secured a patent for a dental applicator; H. B. Gregory, of Newberry, Mich., an artificial tooth for bridgework; V. E. Barnes, of Cleveland, for a new orthodontic appliance; T. H. Whiteside, of Youngstown, Ohio, an artificial tooth fastening for bridgework.

---

New Orthodontic Society.—The alumni of the Angle School for Orthodontia have recently organized a society. Its purpose is to perpetuate the spirit and work of the Angle system of orthodontia and the school in reducing this art to a science. Its first President is Dr. M. Dewey, of Kansas City. It expects to meet annually.

---

Dental Supply Dealers Exhibit.—At Chicago, on March 27 to 30, will be held an extensive exhibit of dental supplies by all the manufacturers and dealers, and it is expected to be the most complete affair of its kind ever undertaken. If you want to see all the new things, make your plans to be there.

—✦—

HISTORY OF DENTISTRY.—Dr. V. Tuerini, of Naples, Italy, has written a history of dentistry from its beginning down to the beginning of the nineteenth century, and it has been translated and will be published in English if 700 subscribers can be had to pay the expense of publication. It will be a book of 500 pages and will cost five dollars. If you want to help out a worthy cause and get an invaluable book, write to Dr. Charles S. Butler, of Buffalo, N. Y., and specify your intention of taking a copy when it shall be published.

—✦—

SOUTHWESTERN MICHIGAN DENTAL SOCIETY.—The next meeting of this vigorous organization will be held April 10th and 11th, at Niles, Michigan. Dr. J. H. Palin, of Grand Rapids, is President this year and Secretary Johnson is out for a good program. We don't know much about Niles or its people, as to what kind of an entertainment they will offer, but we suspect they will know that they have visitors next April, when all Southwestern Michigan, to say nothing of a large Chicago delegation, drops in for a couple of day's work and at least one night of fun. This society has the faculty of getting more fun into a busy meeting than any we ever attended.

—✦—

THE BARNES UNIVERSITY, of St. Louis, announces that Dr. George H. Owen has been appointed Dean, and Drs. O. J. Truth, S. H. Voyles, C. O. Simpson, W. H. Arthur, T. G. Donnell, W. H. Love and C. P. Strawn are new members of the teaching faculty. It is claimed that the recent trouble which precipitated the resignation of the former faculty was caused by the former Dean insisting on the appointment of a demonstrator. The new faculty states that a new building is in process of erection and will be first-class in all its appointments. Sometimes very desirable results come from a first-class quarrel. Let us hope for the best.

—✦—

The third volume of the *Transactions of the Fourth International Dental Congress* is received, and completes the record of this great and historic dental convention. The work is beautifully printed and full of interesting illustrations. We have had little time to read it, but we have no doubt that its editors have done their work faithfully and we shall have much pleasure as well as profit in reading the papers as we did not have in endeavoring to hear them at the meeting.

—✦—

Annual Clinic of the Detroit Dental Society.—This event was held February 12th, and was the most interesting and profitable of the series. The day was given up to clinics in the infirmary of the Dental Department of the Detroit College of Medicine, and at 6 o'clock over one hundred dentists sat down to a very fine banquet, served at the Fellow-Craft Club. After the banquet Dr. G. V. Black gave an illustrated lecture on caries of the teeth with reference to cavity preparation, which was listened to with profound interest. In his usual masterful style he made clear what he means when he speaks of "Extension for Prevention." No one who was present at that lecture and followed the speaker closely will ever be in doubt as why he should extend his cavity margins to safe structure. The magnificent lantern slides were really works of art and excited much favorable comment. Any dental society that can secure Dr. Black for this lecture will do its members one of the greatest favors it can possibly command, and the art of filling teeth will receive a new and needed advancement. The banquet was enlivened by some beautiful singing by Miss Edith Pinckney and Dr. E. B. Spalding, and two fine toastes by Drs. G. C. Bowles and F. W. Holt. This part of the program had to be cut short for lack of time. A very interesting lecture on the color problem in inlay work was given in the afternoon by Dr. J. Q. Byram, of Indianapolis. The clinics were very interesting and held the crowd of visiors because of their value and variety. The following list will give some idea of their character: Dr. S. Straith, "Extraction of Impacted Third Molars and Prolonged Nitrous Oxide Anesthesia." Drs. Shattuck, Oakman and Loucks, "Surgical Cases." Dr. A. P. Biddle, presentation of patients illustrating the "Various Stages of Sphilitic Lesions of the Mouth." J. Q. Byram, Indianapolis, Ind., "Selection and Application of Colors in Inlay Work." W. V. B. Ames, Chicago, Ills., "Mixing and Shading of Cements." G. C. Bowles, "Gold Inlays." C. H. Worboys, Albion, Mich., "Porcelain Crowns and Gold Inlays." E. A. Honey, Kalamazoo, Mich., "Gold Inlays." H. C. Raymond, "Porcelain Inlay for Labial Cavities." W. H. Jackson, Ann Arbor, "Gold Work." F. Ward Holt, Detroit, Mich., "Gold Filling." O. White, Detroit, M. T. Watson, Detroit, H. E. Parshall, Detroit, "Consultation in Orthodontia." E. B. Spalding, C. P. Wood, N. S. Hoff, W. H. VanDeman, G. P. Rogers, "Demonstration of Methods and Results of Prophylactic Treatment." L. M. McPhee, "Steele Detachable Facing." F. Logan, "Crown and Bridge Work." W. C. Herbert, "Porcelain Crowns." M. L. Ward, Ann Arbor, "Alloys." E. Frumveller, "Lingual Attachment for Bridgework." W. H. Graham, "Method of Tightening Loose Dentures." J. M. Thompson, "Porcelain Crowns." H. Kreit, "Continuous Gum." L. I. Luton, "Porcelain Work." E. C. Moore, "Some Historic Porcelain Crowns."

